# Enhancing the Quality and Visibility of African Medical and Health Journals

**DOI:** 10.1289/ehp.12265

**Published:** 2008-12

**Authors:** Thomas J. Goehl, Annette Flanagin

**Affiliations:** Co-director, African Journal Partnership Program, Morrisville, North Carolina, E-mail: tjgoehl@yahoo.com; Co-director, African Journal Partnership Program, Chicago, Illinois

Are most journals published in Africa too weak to be useful to local practitioners, researchers, and policy makers? Might a new method for scholarly communication on the African continent improve the utility of these journals? According to a provocative article published in *Learned Publishing* (Smart 2007), the answer to both questions is yes. Smart argued that the African research and education communities need to rethink their tendency to “slavishly . . . follow the Western model of academic promotion based on publishing in journals.” In an earlier article, Horton (2000a) voiced concerns that researchers, policy makers, and philanthropic organizations in developed countries believe simply providing access to Western information will solve many of the problems of developing nations. On the contrary, he wrote, in Africa “there is already a well-developed local information culture that needs support, not swamping,” noting, moreover, the lack of African journals in MEDLINE (Horton 2000a).

According to a survey conducted in 2005, about 158 medical journals were published in 33 African countries, but most had circulations <1,000, were published ≤4 times per year, and were excluded from major bibliographic indexes (Siegfried et al. 2006). African Journals Online, an online repository of African scholarly abstracts hosted by the International Network for the Availability of Scientific Publications (INASP), lists 111 health and medical journals from 18 African countries in 2008 (African Journals Online 2008). However, compared with those of other continents, African medical and health journals continue to be poorly represented in international indexing services: among 5,000 journals indexed in MEDLINE, 38 are from Africa (13 countries), and among 6,700 journals in the Institute for Scientific Information’s Science Citation Index, only 20 are from Africa (4 countries), including just 1 medical journal.

Even within Africa, there is a disparity in research publication, with South Africa, Egypt, and Nigeria producing 60% of the total number of articles indexed by PubMed between 1996 and 2005 (Uthman and Uthman 2007). Moreover, there are gaps in research information published in leading Western and Northern journals on conditions and diseases that are most relevant to low-income countries in Africa (Horton 2000b).

Thus, despite the recognized benefits of medical journals to health practitioners (Gross 2000; Lamas 1992), Africa’s medical journal and research production and distribution are low, and as a result, research from Africa is not readily available to colleagues on the continent or in the international scientific community. Quality journals are needed throughout Africa to help raise the visibility of African science to researchers, practitioners, and policy makers in countries within Africa, especially sub-Saharan Africa, and to the wider international community (Ofori-Adjei 2006).

Both Smart (2007) and Horton (2000a, 2000b) provide insights into the troubling issue of scientific and medical communications within Africa. Although there is no doubt that many of the medical and health journals published in Africa are weak, it would not be appropriate to declare them moribund and give up on the traditional peer-reviewed journal model for these medical, health care, and research communities (Ofori-Adjei 2006). In fact, substituting another communication vehicle for this specific subset of journals would need to be undertaken with extreme caution, if at all. Scientific journals serve not only as gatekeepers but also as gateways for the dissemination of quality research, playing an essential role in the transfer of knowledge among practitioners. Such information, published by medical and health journals, is used by medical and public health professionals and must be vetted thoroughly to avoid negatively impacting the practice of medicine. Determining how to provide the needed support to help improve these journals must be carefully considered (Tucker 1999) and must remain respectful of the existing local information culture.

One attempt to provide the needed support for African journals that face such daunting problems (Goehl 2007) is a program called the African Journal Partnership Project (AJPP), initiated in 2004 by the U.S. National Library of Medicine (NLM), the John E. Fogarty International Center for Advanced Study in the Health Sciences, and the U.S. National Institute of Environmental Health Sciences and administered by the Council of Science Editors (CSE). This project partners African journals with leading U.S.- and European-based journals (Nading 2005; Tillett 2005).

In the initial phase of the project, four African journals participated: *African Health Sciences* was paired with *BMJ*, *Ghana Medical Journal* with *The Lancet, Malawi Medical Journal* with *JAMA*, and *Mali Médical* with *Environmental Health Perspectives* (*EHP*) and the *American Journal of Public Health*.

The objectives of these journal partnerships are to strengthen the four African health journals such that they could provide training for African health researchers, improve the quality and visibility of their research, and make the journals a better resource for local researchers and policy makers. To ensure the partnership program is on course for reaching these goals, formal evaluations have been conducted for each African partner journal to monitor ongoing success and identify achievements as they relate to the objectives. Another objective of the AJPP is for its African members to become regional leaders and share their acquired knowledge and experience with other editors and journals on the continent.

In its initial four years, the AJPP has been quite successful in meeting its original goals. Editors have organized and attended workshops on journal management including training on improving business plans and developing strategic plans for effective, sustainable publishing operations. Authors, editors, clinicians, and scientists have also received training on better ways to communicate research and write for publication, as well as training in statistics, data presentation, and data interpretation. The African partner journals are now all publishing regularly in print and/or online. Two of the journals (*Mali Médical* and *Malawi Medical Journal* ) have dedicated websites, and three (*African Health Sciences*, *Malawi Medical Journal*, and *Mali Médical* ) have been accepted into MEDLINE. Because of that success, the NLM added two more partnerships to the project this year, pairing the *Medical Journal of Zambia* with the *New England Journal of Medicine* and the *Ethiopian Journal of Health Sciences* with the *Annals of Internal Medicine*.

Scientific journals serve not only as gatekeepers but also as gateways for the dissemination of quality research, playing an essential role in the transfer of knowledge among practitioners.

Given the state of publishing in Africa, the challenge of elevating the relevance and usefulness of the continent’s medical/health journals is enormous. Leveraging resources has been a key feature of the AJPP, which itself has successfully leveraged its NLM funding by attracting additional support from its international journal partners as well as from SPi Technologies, ScholarOne, the World Health Organization Special Programme for Research and Training in Tropical Diseases (WHO/TDR), INASP, and CSE.

SPi Technologies is providing complimentary services to convert the African journal partner print issues into XML format, which are compatible for submission to PubMed Central. This service started with the 2005 issues.

ScholarOne provides its web-based manuscript submission and review system (Manuscript Central) without charge to the three African journal partners that are published in English (*Mali Médical* is a francophone publication). The system was fully launched in 2007 for the *Malawi Medical Journal* and in 2008 for *African Health Sciences* and *Ghana Medical Journal.* Each of these three African journal partners is receiving training in the operation of the system.

WHO/TDR has provided travel funds for non-AJPP African journal editors belonging to the Forum for African Medical Editors (FAME) to attend workshops conducted by partner journals. This provision helps to spread the positive effect of the partnership to additional African editors, journals, and countries.

INASP helped support a workshop on journal business operations, furthering the partnership’s goal of establishing plans for journal sustainability.

In addition to helping with administrative logistics for the partnership, CSE has provided complimentary membership to the African journal editor partners and has waived registration fees for those attending the council’s annual meeting and workshops. The African editors have become active council members, participating in CSE committees and speaking at annual meetings.

Our experience has shown that to have a successful project, the partners must be selected carefully, matching the interests of both journals. The importance of having dedicated, enthusiastic individuals cannot be overstated. There are also concerns about communication, respect, and understanding of cultural differences. Electronic communication is efficient given the geographic distances between the partners, but face-to-face meetings are of utmost importance, and the earlier they occur in the partnership, the better. Trust develops when both partners have shown respect for each other’s perspective, assistance is given based on the needs expressed, and learning and knowledge transfer are multidirectional.

Smart’s concerns about the status of African journals (Smart 2007) and Horton’s admonition to avoid swamping the existing information culture (Horton 2000a) must be taken seriously, and the AJPP gives strong indication that it is meeting both concerns at once. From our experience, African journal editors have an energy and dedication equaling that of their Western counterparts. Although the needs of the African journals are great, we believe partnerships such as the AJPP will continue to help African medical and health journals achieve success by listening to their editors and providing the proper assistance with humility. Ultimately, their success will benefit not only the peoples of Africa but also those of the developed world.

## Figures and Tables

**Figure:**
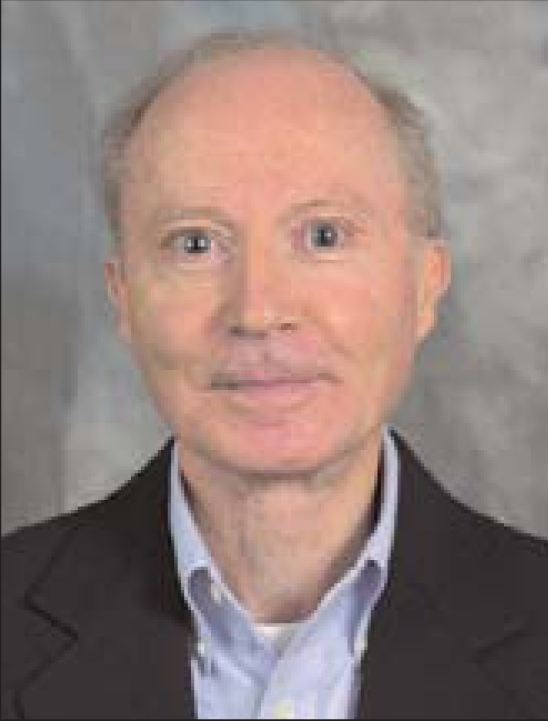
Thomas J. Goehl

**Figure:**
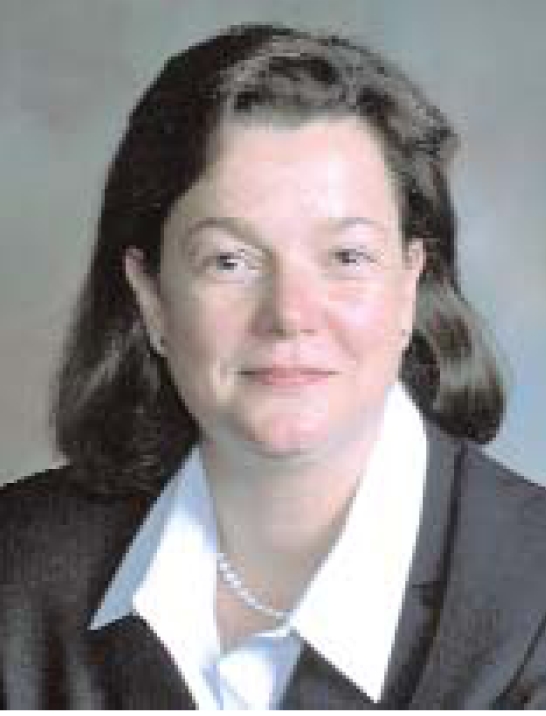
Annette Flanagin

## References

[b1-ehp-116-a514] African Journals Online (2009). Homepage.

[b2-ehp-116-a514] Goehl TJ (2007). Access denied. Environ Health Perspect.

[b3-ehp-116-a514] Gross GP, Steiner CA, Bass EB, Powe NR (2000). Relation between prepublication release of clinical trial results and the practice of carotid endarterectomy. JAMA.

[b4-ehp-116-a514] Horton R (2000a). Development aid: manna or myth?. Lancet.

[b5-ehp-116-a514] Horton R (2000b). North and South: bridging the information gap. Lancet.

[b6-ehp-116-a514] Lamas G, Pfeffer M, Hamm P, Wertheimer J, Rouleau J, Braunwald E (1992). Do the results of randomized clinical trials of cardiovascular drugs influence medical practice?. N Engl J Med.

[b7-ehp-116-a514] Nading T (2005). CSE helps manage partnerships to enhance African journals. Science Editor.

[b8-ehp-116-a514] Ofori-Adjei D, Antes G, Tharyan P, Slade E, Tamber PS (2006). Have online international medical journals made local journals obsolete?. PLoS Med.

[b9-ehp-116-a514] Siegfried N, Busgeeth K, Certain E (2006). Scope and geographical distribution of African medical journals active in 2005. S Afr Med J.

[b10-ehp-116-a514] Smart P (2007). Journals—the wrong model for Africa?. Learned Publishing.

[b11-ehp-116-a514] Tillett T (2005). Global collaboration gives greater voice to African journals. Environ Heath Perspect.

[b12-ehp-116-a514] Tucker N, Munck R, O’Hearn D (1999). The myth of development: a critique of a Eurocentric discourse. Criticial Development Theory.

[b13-ehp-116-a514] Uthman OA, Uthman MB (2007). Geography of Africa biomedical publications: an analysis of 1996–2005 PubMed papers. Int J Health Geogr.

